# Supplementary material for: A Critical View of the Application of the APEX Software (Aqueous Photochemistry of Environmentally-Occurring Xenobiotics) to Predict Photoreaction Kinetics in Surface Freshwaters
Source: Molecules. 2019 Dec 18;25(1):9. doi: 10.3390/molecules25010009 (PMC7017383; doi:10.3390/molecules25010009)
Supplement: Supplementary file 1 [file molecules-25-00009-s001.zip › 3.2.4_gcc-4.4.0.zip!/3.2.4_gcc-4.4.0/3.2.4_gcc-4.4.0/doc/octave/html/interpreter/Current-Working-Directory.html]

Current Working Directory - Untitled


Next: Password Database Functions,
Previous: Environment Variables,
Up: System Utilities


---

### 34.8 Current Working Directory

— Command: **cd** dir  
— Command: **chdir** dir  
> Change the current working directory to dir. If dir is
> omitted, the current directory is changed to the user's home
> directory. For example,
>
> ```
>           cd ~/octave
> ```
>
> Changes the current working directory to ~/octave. If the
> directory does not exist, an error message is printed and the working
> directory is not changed.
>
>
>
> **See also:** mkdir, rmdir, dir.

— Command: **ls** options  
> List directory contents. For example,
>
> ```
>           ls -l
>                -| total 12
>                -| -rw-r--r--   1 jwe  users  4488 Aug 19 04:02 foo.m
>                -| -rw-r--r--   1 jwe  users  1315 Aug 17 23:14 bar.m
> ```
>
> The `dir` and `ls` commands are implemented by calling your
> system's directory listing command, so the available options may vary
> from system to system.
>
>
>
> **See also:** dir, stat, readdir, glob, filesep, ls\_command.

— Function File: [old\_cmd = **ls\_command** (cmd)  
> Set or return the shell command used by Octave's `ls` command.
> The value of cmd must be a character string.
> With no arguments, simply return the previous value.
>
>
>
> **See also:** ls.

— Function File: **dir** (directory)  
— Function File: [list] = **dir** (directory)  
> Display file listing for directory directory. If a return
> value is requested, return a structure array with the fields
>
> ```
>           name
>           bytes
>           date
>           isdir
>           statinfo
> ```
>
> in which `statinfo` is the structure returned from `stat`.
>
> If directory is not a directory, return information about the
> named filename. directory may be a list of directories
> specified either by name or with wildcard characters (like \* and ?)
> which will be expanded with glob.
>
> Note that for symbolic links, `dir` returns information about
> the file that a symbolic link points to instead of the link itself.
> However, if the link points to a nonexistent file, `dir` returns
> information about the link.
>
>
>
> **See also:** ls, stat, lstat, readdir, glob, filesep.

— Built-in Function: **pwd** ()  
> Return the current working directory.
>
>
>
> **See also:** dir, ls.
